# Therapeutic application of nano-encapsulated pomegranate peel extract attenuated DSS-induced colitis: Antioxidant and anti-inflammatory role and reduction of exaggerated response of endoplasmic reticulum stress

**DOI:** 10.1371/journal.pone.0323605

**Published:** 2025-05-13

**Authors:** Abdallah Tageldein Mansour, Safaa I. Khater, Hemmat M. Eissa, Helal F. Al-Harthi, Areej A. Eskandrani, Mohammed Ageeli Hakami, Wafa S. Alansari, Amirah Albaqami, Hanan M. Alharbi, Tarek Khamis, Doaa Ibrahim

**Affiliations:** 1 Animal and Fish Production Department, College of Agricultural and Food Sciences, King Faisal University, Al-Ahsa, Saudi Arabia; 2 Department of Biochemistry, Faculty of Veterinary Medicine, Zagazig University, Zagazig, Egypt; 3 Department of Biology, Turabah University College, Taif University, Taif, Saudi Arabia; 4 Chemistry Department, College of Science, Taibah University, Medina, Saudi Arabia; 5 Department of Clinical Laboratory Sciences, College of Applied Medical Sciences, Shaqra University, Al-Quwayiyah, Riyadh, Saudi Arabia; 6 Biochemistry Department, Faculty of Science, University of Jeddah, Jeddah, Saudi Arab; 7 Department of Clinical Laboratory Sciences, Turabah University College, Taif University, Taif, Saudi Arabia; 8 Department of Biology, College of Science, Princess Nourah bint Abdulrahman University, Riyadh, Saudi Arabia; 9 Department of Pharmacology, Faculty of Veterinary Medicine, Zagazig University, Zagazig, Egypt; 10 Department of Nutrition and Clinical Nutrition, Faculty of Veterinary Medicine, Zagazig University, Zagazig, Egypt; University of Cagliari: Universita degli Studi Di Cagliari, ITALY

## Abstract

The medicinal application of pomegranate peel extract enriched with polyphenols (PPE) as a therapeutic strategy for managing inflammatory bowel diseases (IBD) is still limited. Integrating pomegranate peel extract (PPE) into an effective nanocarrier system could enhance its mechanistic actions, potentially aiding in the remission of colitis. Therefore, this approach aimed to enhance PPE’s stability and bioavailability and investigate mitigating impact of pomegranate peel extract-loaded nanoparticles (PPE-NPs) in a colitis model. Colonic injury was induced by 5% dextran sulfate sodium (DSS) and efficacy of disease progression after oral administration of PPE-NPs for 14 days was assessed by evaluating clinical signs severity, antioxidant and inflammatory markers, expressions of endoplasmic reticulum associated genes and histopathological and immunostaining analysis in colonic tissues. Clinical signs and disease activity index were effectively reduced, and the levels of fecal calprotectin were decreased in groups treated with PPE-NPs compared to DSS group. The colitic group showed a significant increase (P < 0.05) in C-reactive protein (CRP) and myeloperoxidase (MPO) and nitric oxide (NO) (35.60, 163.30 and 280 nmol/g tissue respectively) and higher expression (P < 0.05) *IL-17*, *TNF-α*, and *IL-1β* (increased up to 2.99, 4.36 and 4.90 respectively unlike PPE-NPsIII that recorded reduced levels of CRP, MPO and NO (8,96, 78.30 and 123 nmol/g tissue respectively) and much lower (P < 0.05) levels of IL-17, TNF-α, and IL-1β expression (decreased to 1.23, 1.69 and 1.64, respectively). The most improvement of colon damage PPE-NPsIII group was also associated with the reduction MDA level (P < 0.05) (decreased to 21.60 vs 90.65 in DSS non treated group). The highest glutathione peroxidase, superoxide dismutase and catalase activities were noted in PPE-NPsIII received group (42.60, 50.30 and 62.70 U/mg). Notably, prominent free radical scavenging activities were noticed in group received 150 mg/kg of PPE-NPs as supported by higher scavenging of 1,1-diphenyl-2-picrylhydrazyl (9.85 mg/g) and 2,2-azinobis-(3-ethylbenzothiazoline-6-sulfonic acid tested radicals (19.98 mg/g). Balancing between endoplasmic reticulum stressors (ERS), inflammation and autophagy was prominently noted in group treated with 150 mg/kg of PPE-NPs. These findings were supported by subsiding the excessive expression of ERS related genes (*CHOP*, *JUNK*, *ATF6*, *BIP*, and *Elf*-2) and immunostaining expression regulation of key markers regulating autophagy (Beclin-2) in this group. The histopathological changes in the colon were less severe in the PPE-NPs received groups (especially at the level of 150 mg/kg) compared to DSS group. Collectively, these findings suggest that the nanoencapsulation of PPE enhances its effectiveness in promoting recovery of colonic tissue damage and achieving remission of colitis.

## 1. Introduction

Ulcerative colitis and Crohn’s disease, which are characterized by persistent inflammation of the gastrointestinal tract (GIT), are classed as Inflammatory Bowel Disease (IBD) with high prevalence globally [1,2]. The development of IBD is multifactorial and establishes a complex interplay between variety of genetic, immunological, and environmental triggers that interrupt the gut homeostasis like oxidative stress with election of intestinal inflammatory responses [3]. This active inflammation is coupled with the production of reactive species associated with oxidative stress which considered as is a key feature of IBD [4]. Excessive reactive oxygen species (ROS) generation and interrelated products can also be harmful as their incessant release in the local microenvironment of inflamed mucosal lesions results in widespread molecular and cellular damage, prolonging of intestinal inflammation and destruction [5]. Therefore, there is a continuous search for immunomodulatory and antioxidants supplements that promote gastrointestinal health, and it has been suggested that polyphenols present in plant-based meals may be useful in achieving this goal [6–8]. Food sources that enriched in polyphenols have garnered attention for their advantageous impact on human well-being, supported by evidence of their antioxidant, anti-cancer [9–11], cardio-protective, and anti-inflammatory properties [12,13]. Additionally, *Lactarius deliciosus*, a widely recognized mushroom known for its delightful taste and texture, has demonstrated immunomodulatory activity in vitro and can modulate the gut microbiome [14]. *Punica granatum,* also referred to as pomegranate, is acknowledged for its health advantages, including antioxidant, antibacterial, anti-inflammatory, and anti-cancer properties [15,16]. The peels, a primary byproduct of pomegranate juice manufacturing, contains a contain distinct amounts of flavonoids, specifically anthocyanins and higher concentration of hydrolysable tannins, specifically punicalagins [17]. Punicalagins are a group of esters composed of gallic acid and glucose and are well-known for their antioxidant and anti-inflammatory effects [18–20]. Pomegranate seed extract can relieve colitis via modulation of oxidative stress and inflammation [21]. It was documented that suggests that Pomegranate extract, via its anti-inflammatory and antioxidant functions, may provide a hopeful complementary treatment option, remarkably in healing inflammatory bowel [22]. Additionally, pomegranate components have a protective function against colitis via their anti-inflammatory effect [23]. Despite these advantages, the polyphenolic content in pomegranate seed extract are sensitive to various environmental conditions such as light, oxygen, pH, etc., and encapsulation is a promising technique that can protect them in such conditions and improve their activity [24]. Additionally, encapsulation of pomegranate peel extract bioactive components can preserve and enhance their physicochemical functionalities and enhances controlled release of them [25]. The benefits of encapsulating bioactive compounds in nanoparticles include: (i) safeguarding the compounds from negative environmental influences, thereby prolonging the shelf life of unstable compounds [26]; (ii) creating nanomaterials that enable targeted delivery, controlled release, and enhanced therapeutic and functional effects; (iii) enhancing the physical properties and ease of handling of the core bioactive materials; and to trap natural substances within its structure [27] and thus enhancing their performance and compatibility [28]. Additionally, it possesses several benefits, including non-toxicity, biocompatibility, and antibacterial capabilities [29] rendering its application in biomedical treatments [29]. Currently, there is a lack of research examining the distinct attributes of CSNPs that incorporate pomegranate peel extract (PPE). Hence, the aim of this study was to verify the supportive therapeutic potential derived from PPE-NPs for ulcerative colitis in human via reducing the oxidative and ER stress along with their subsequent effects, including excessive inflammatory responses and autophagy.

## 2. Materials and methods

### 2.1. Preparation of pomegranate peel extract

Complete and undamaged Egyptian Baladi pomegranate fruits were purchased from a nearby market when they were at their peak of ripeness in late September. The maturity of the fruits was evaluated based on their appearance, including the color of the external skin and juice. The fruit was peeled by hand, washed and then the peels were air dried at room temperature (in shade) for seven days. The peels were ground into a powder and sieved through a 700 m mesh. For 3 days at room temperature in the dark, 500 g of peel powder was submerged in a 2.5 L hydromethanol solution (with a methanol: water ratio of 4:1). After that, the solids were filtered out by gently shaking the suspension using a vacuum rotary evaporator (Heidolph Instruments Co., model VV 2000, Schwabach, Germany) set to 40 °C, the methanol was extracted from the residual extract. After collecting the extract, it was dried to 20% moisture content at room temperature (23 °C) in the dark for 5 days before being frozen at 18 °C for later use [30]. Fraction of PEE contents was done by High-performance liquid chromatography (HPLC) (Agilent 1100, Merck KGaA, Darmstadt, Germany) (Table 1).

**Table 1:** Flavonoid and phenolic compounds of pomegranate peel extract analyzed by HPLC.

Flavonoids compounds	(mg/kg)	Phenolic compounds	(mg/kg)
Quercetrin	33.98	Ellagic	122.87
Rutin	2.87	Cinnamic	26.87
Naringin	9.66	3,4,5-methoxycinnamic	1.23
Quercetrin-3-O-glucose	2.12	Coumarin	8.54
Apigenin-7-glucose	7.14	Salicylic	1.25
Kaempferol	0.88	Catechol	62.33
Apegnin	1.15	Catechein	31.87
Hespirtin	5.31	Caffeine	12.76
Kaemp.3-(2-p-coumaroyl) glucose	8.43	Vanillic	7.43
Acacetin7 neo-hesperside	3.68	Ferulic	4.90
Luteo.7-glucose	6.04	Gallic	25.90
Apig.6-rhamnose galactose	7.22	Pyrogallol	325.70
Apig.6-arbinose 8-galactose	3.17	4-Amino-benzoic	0.78
Apig.7-O-neohesperidoside	3.09	Protocatchuic	18.64
Quercetin	1.98	Chlorogenic	17.12
Acacetin7 neo hesperside	4.13	Rosmarinic	12.30
Rhamentin	2.57	Alpha-coumaric	2.94
		Iso-ferulic	0.93
		p-coumaric	0.76
		Caffeic	4.39
		Benzoic	6.54

### 2.2. Determination of total polyphenol and flavonoid content in PPE.

Total polyphenol content was estimated as described by Singleton and Rossi [31] using Folin–Ciocalteu reagent and gallic acid (as standard) with slight modifications (Singleton & Rossi, 1965). Briefly, PPE (1 mg) was dissolved in distilled water (5 mL, in triplicate) then sodium carbonate (1 mL 20%; w/v) and Folin–Ciocalteu reagent (1 mL) were added then thoroughly mixed and transported to water bath for 30 min at 40 °C in standing position. The color absorbance was assessed at 765 nm via UV-V spectrophotometer. Total polyphenol content was expressed as milligrams of gallic acid equivalent per gram of dried extract (mg GAE/g E). Regarding total flavonoid content, their contents were evaluated according to method [32], as brief, 0.5 mL of PPE was mixed with 10% aluminium chloride (0.1 mL), methanol (1.5 mL), 1M potassium acetate (0.1 mL) and 2.8 mL distilled water in the test tube then incubated at room temperature for 30 min. The absorbance was measured at 415 nm, and the total flavonoid content was calculated as mg quercetin equivalents (QE/L).

### 2.3. Preparation and characterization of PPE-loaded CSNPs

**Fig 1:**
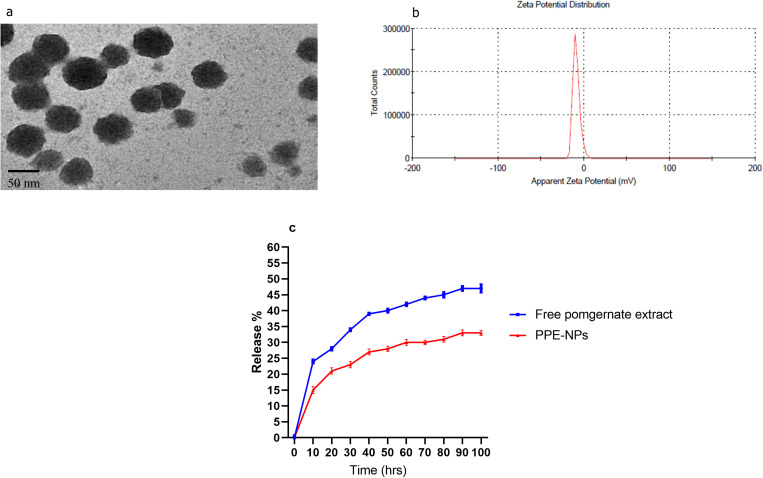
Transmission electron microscopy. (a), Zeta potential and particles size (b) of pomegranate peel extract encapsulated chitosan nanoparticles (PPE-NPs).

Fully stocked with protective equipment to make chitosan nanoparticles (CSNPs), we used sodium tripolyphosphate (STPP) as a crosslinking agent in an ionic gelation process. LMW chitosan (0.2 g) was added to 40 mL acetic acid (1% v/v) to achieve a 0.5% w/v con centration, and it was then held overnight under magnetic stirring at ambient temperature to get a transparent solution, and its pH was adjusted to 4.6 using a 1 M NaOH. The resulting chitosan solution was filtered using a syringe with a 0.45 m filter. Magnetic stirring (at 500 rpm) was applied to a chitosan solution containing PPE for 60 minutes at room temperature (23 °C). After preparing a stock solution of STPP in double-distilled water with a concentration of 0.2% (w/v), filtering it through a 0.22 m syringe filter, and adjusting the pH to 5.6 with one M HCL, the solution was kept at 4 °C until use. CSNPs were made by adding 10 milliliters of cold STPP solution to 10 milliliters of chitosan solution containing PPE while stirring at 1400 revolutions per minute and letting the mixture sit in an ice bath for 50 minutes. After a suspension was obtained, it was centrifuged at 9000 rpm for 30 minutes [33]. The supernatant was removed for further study, and the CSNP pellets that had settled to the bottom were washed multiple times in deionized water. For 20 minutes (sonication/resting cycles of 1 minute), moist pellets were sonicated with a probe sonicator (VCX 130, Vibra Cell Sonics, Newtown, CT, USA) in an ice-bath to achieve a homogeneous suspension. CSNPs were then lyophilized at − 60 °C for 72 hours and stored refrigerated for subsequent analysis.The characterization of produced PPE-loaded CSNPS was conducted using transmission electron microscopy (TEM) (JEOL JEM-2100; JEOL Ltd, Tokyo, Japan) with the assistance of digital micrograph and soft image viewer software, and the analysis of zeta potential dispersion by using dynamic light scattering analysis with the Malvern Zetasizer, Nano ZS (Fig 1a and b). The in vitro release of PPE from PPE-NPs was investigated using a dialysis bag (MW = 12 kDa) against phosphate-buffered saline (PBS) at pH = 7.4 at 37°C according to [34]. The *in vitro* release assay was performed at different intervals (0–100 minutes, Fig 1C) and the amounts of therapeutic agents in the examined samples were analyzed via the established HPLC assay.

### 2.4. Ethics statement, animal model and induction of colitis

The study was conducted in accordance with the recommendations in the guide for care, the arrival guidelines and use of laboratory animals of Zagazig University_ Institutional Animal Care and Use Committee (ZU_IACUC) with approval number (ZU-IACUC/2/F/165/2023). All surgery was done under intraperitoneal Thiopental sodium (50 mg/kg, 0.1 ml/100 g/kg) for anesthesia prior to animal euthanasia.

A total of 50 male albino rats of the Sprague-Dawley strain (aged 7–8 weeks, 200 ± 20 g), were purchased from the Animal House at the Faculty of Veterinary Medicine, Zagazig University. The rats were divided into five group using a random allocation method, with each group consisting of 10 rats (n = 10) as follows: control (non colitic group), DSS group (animals that received 5% DSS and not subjected to any form of therapy), PPE-NPsI group (animals that received 5% DSS and subjected to 50 mg/kg of PPE-NPs, PPE-NPsII group (animals that received 5% DSS and subjected to 100 mg/kg of PPE-NPs, PPE-NPsIII group (animals that received 5% DSS and subjected to 150 mg/kg of PPE-NPs. DSS was administered a 5% w/v concentration of (Mw = 7000–20,000; product No. 51227-100G, Sigma-Aldrich, Shanghai, China) in the drinking water of the animals for 7 days. The period of treatment by different concentrations of PPE-loaded NPS was extended for 14 days. During the experimental period no deaths were recorded, and daily observations were made on physical activity, clinical symptoms and daily monitoring of body weight was conducted in conjunction with the assessment of colitic illness activity. The assessment of stool consistency, namely the presence of water or blood, was used to establish the severity of clinical symptoms. The colon and spleen were dissected in order to assess the length of the colon and the weight of the spleen. The assessment of gross rectal bleeding and stool consistency was conducted in accordance with established protocols.

### 2.5. Sampling

At the end of the experimental period, all rats were anesthetized with intravenous ketamine hydrochloride (30 mg/kg body weight) to minimize suffering and distress, and were subsequently euthanized through cervical dislocation. Blood samples (n = 5/group) were obtained from the vena cava, for hematological profiling in heparinized tubes, and for coagulation, and after that subjected to centrifugation at a speed of 3,000 revolutions per minute for 15 minutes. The serum samples were stored at a temperature of -20 °C till they were further used. The fecal marker was evaluated using fecal samples that were collected. Following a treatment period of 30 days, the rats were euthanized, and the colon tissues were then isolated. The colon samples (the distal 10 cm section of the colon) were divided into two portions. The first portion was enveloped in aluminum foil and promptly placed in a liquid nitrogen container to facilitate rapid freezing of the tissue and reduce the activity of endogenous RNase. This was done to enable real-time PCR analysis and immunohistochemistry evaluation. The second portion was preserved in a 10% formalin solution at ambient temperature for 24 hours. Subsequently, it underwent staining with hematoxylin-eosin (H&E) in order to facilitate histological examination of the colon components.

### 2.6. Disease activity index

The disease activity index (DAI) was employed on a daily basis to assess the severity of the ailment as presented in Table 2. The DAI encompassed parameters such as weight loss, stool consistency, rectal hemorrhage, and general animal condition. The rats were assigned a numerical score on a scale of 0 (indicating no impact) to 3 (indicating a significant impact). The scores were subsequently aggregated to obtain a composite DAI [35].

**Table 2:** Scoring of Disease Activity Index (DAI).

	Body mass loss (%)	Stool consistency	Rectal bleeding
Scores	0 = None	0 = Normal consistency	0 = Negative
	1 = 0.1-5%		
	2 = 5-10%	2 = Loose stool	2 = rectal occult blood
		3 = 10-20%	
	4 = > 20%	4 = Diarrhea	4 = Gross bleeding

### 2.7. Biochemical analysis

The determination of red blood cell (RBC) counts was performed according to standard clinical methods. The quantification of hemoglobin (Hb) concentration was performed in accordance with the method described Van Kampen & Zijlstra, [36]. The serum activity of alanine amino transferase (ALT) and aspartate amino transferase (AST) were determined colorimetrically by (ALT Assay Kit abcam, Cat. No-ab105134; AST Assay Kit abcam, Cat. No-ab105135, USA) according to manufacture instructions respectively [37]. Serum creatinine and urea were assayed enzymatically using enzymatic Kit (Creatinine Assay Kit abcam, Cat. No- ab65340; Urea Assay Kit abcam, Cat. No- ab83362, USA) according to manufacture instructions [38,39].

### 2.8 Assessment of oxidant stress markers and cytokines in colonic tissues by Enzyme-Linked Immunosorbent Assay

The TAC levels were determined using OxiSelect™ TAC Assay Kit (MyBioSource, Catalog No: MBS841488, USA) as per the manufacturer’s instructions [40]. Catalase (CAT, ELISA Kit MyBioSource, Catalog No: MBS726781, USA), glutathione peroxidase (GPx, ELISA Kit MyBioSource, Catalog No: MBS744364, USA), MDA (ELISA Kit MyBioSource, Catalog No: MBS268427, USA) and superoxide dismutase (SOD, ELISA Kit MyBioSource, Catalog No: MBS036924, USA). Interferon gamma (INFγ, ELISA Kit MyBioSource, Catalog No: MBS267008, USA). Interlekin-6 (IL6, ELISA Kit MyBioSource, Catalog No: MBS355410, USA) as per the manufacturer’s instructions.

### 2.9. Assessment of myeloperoxidase levels in the colonic tissues

A total of 10 μL of homogenized colon tissue was combined with 80 μL of a solution containing 0.75 mM H₂O₂ and 110 μL of a 3,3′,5,5′-Tetramethylbenzidine (TMB) solution. The TMB solution consisted of 2.9 mM TMB in a mixture of 14.5% Dimethyl sulfoxide and 150 mM sodium phosphate buffer at a pH of 5.4. The measurement of absorbance was promptly taken at a wavelength of 450 nm, with a reference wavelength of 620 nm, using a microplate reader. Subsequently, the specimen was subjected to incubation at a temperature of 37°C for 15 minutes. Subsequently, to terminate the reaction, 50 μL of a 2 M H₂SO₄ solution was added, and the absorbance was measured using spectrophotometry at a wavelength of 450 nm. In the experiment, a total volume of 10 μL of horseradish peroxidase (HRP) was used at concentrations of 2.5 mU/mL and 25 mU/mL. The measurement of MPO activity was determined by calculating the absorbance difference corresponding to the HRP standard curve and the data was presented in the form of mU/mL [41].

### 2.10. Determination of nitric oxide and C-reactive protein levels in the colonic tissues

The concentrations of nitric oxide in the colon were assessed using the Griess technique. A total of 50 μL of colon tissue that had been homogenized was mixed with 50 μL of Griess reagent, which consisted of 0.1% sulphanilamide, 3% phosphoric acid, and 0.1% naphthyl ethylenediamine. The mixture was then kept at room temperature for 10 minutes, while being shielded from light. Subsequently, the measurement of absorbance was conducted at a wavelength of 540 nm using a microplate reader. The concentration of nitrite was determined by utilizing the standard curve [42]. The evaluation of C-reactive protein (CRP) levels was conducted in accordance with the criteria provided by a commercial kit (AG723-M; Sigma-Aldrich, USA) [43].

### 2.11. Determination of fecal calprotectin

Stool samples were collected and homogenized for 25 minutes after being combined with extraction buffer. The homogenate was then centrifuged for 20 minutes, and the resulting supernatant was stored in the freezer at 20 °C. Briefly, 100 mL of diluted supernatants were put into the microtiter plate wells then incubated for 45 min at room temperature on a shaker (600 rpm), and subsequently washed three times with washing solution. In the next step, purified rabbit anti-calprotectin antibodies conjugated with alkaline phosphatase were added and incubated at room temperature for 45 min, then enzyme substrate was added after washing the wells three times. Calprotectin concentrations were estimated from the standard curve attained with kits standards of ELISA (Thermo Fisher; MA5–12213) as described by [44].

### 2.12. Determination of scavenging activities

ABTS [2,2-azinobis-(3-ethylbenzothiazoline-6-sulfonic acid)] assay: The total antioxidant capacity of homogenized colonic tissues was analyzed by Trolox-equivalent antioxidant capacity (TAC) assay [45]. Briefly, the reaction between ABTS (7 mM in H2O) [2,2-azinobis-(3-ethylbenzothiazoline-6-sulfonic acid)] with an equal volume of potassium persulfate (4.9 mM in H2O) was allowed at room temperature for 12–16 h in the dark to stimulate the formation ABTS+ radical cation formation. Then ABTS radical solution was diluted via thoroughly blending with ethanol (96%) to achieve an absorbance of 0.7 at 734 nm via a spectrophotometer. In the next step, samples of about 10 μL were added to dimethyl sulfoxide then to ABTS radical solution (190 μL) in a 96-well microplate, then incubated at room temperature for 30 min. and absorbance were read at 734 nm.

DPPH assay: The scavenging activity of the colonic samples was analyzed by 1,1-diphenyl-2- picrylhydrazyl radical (DPPH) [45]. Briefly, 190 μL of 0.1 mM DPPH solution in MeOH were mixed with 10 μL of samples in dimethyl sulfoxide in wells of a 96-well plate then thoroughly shaken and incubated at room temperature for 30 min in the dark place. After that, the absorbance was read at 517 nm by spectrophotometer. The ability to scavenge the DPPH radical was expressed as mg per g of tissue.

### 2.13. Gene expression

A quantity of 30 mg of colonic tissue using Trizol (Invitrogen; Thermo Fisher) was utilized to isolate total RNA from tissue and the NanoDrop® ND-1000 was utilized. The cDNA was combined with an RH HiSenScriptTM (cDNA Synthesis Kit). The real-time RT-PCR procedure was conducted in accordance with the guidelines provided by the manufacturer using the MX3005P Real-Time PCR (Agilent Stratagene, USA) and the 5X HOT FIRE Pol EvaGreen qPCR Mix Plus (Solis BioDyne, Tartu, Estonia). The experimental protocol involved conducting the polymerase chain reaction with specific parameters. Initially, the reaction was subjected to an initial denaturation step at a temperature of 95°C for 12 minutes. Subsequently, denaturation was carried out at 95 °C for 20 seconds, repeated for a total of 40 cycles. Following this, annealing was performed at a temperature of 60 °C for 30 seconds. Subsequently, extension was carried out at 72°C for 30 seconds. Finally, a melting curve analysis was conducted. The expression level of the target genes was normalized by comparing them to the housekeeping gene β-actin using the RNA formula ^2-ΔΔ^ct [46]. The specific primer sequences for the target genes are provided in Table 3.

**Table 3:** Primers sequences used in this study.

Gene	Primers	Gene bank accession number
*TNF-α*	F 5′-GGGGCCACCACGCTCTTCTGTC-3’ R, TGGGCTACGGGCTTGTCACTCG-3’	NM_012675.3
*IL-β*	F 5′- CAGGACGAGGACCCAAGAAC -3’ R 5’- TCAGACAGCACGAGGCATTT -3’	XM_032902343.1
*IL-10*	F 5′- CCTCTGGATACAGCTGCGAC-3’ R 5’- CGCCGGGTGGTTCAATTTTT-3’	XM_032915519.1
*VEGF*	F 5′- TTTTTGGGGAGCCTCAGGAC -3’ R 5’- GGAGGAGAGAGCTTGATGGC -3’	NM_031836.3
*CCR7*	F 5′- TGGTCATTTTCCAGGTGTGCT-3’ R 5′-CAGGCCTTAAAGTTCCGCAC-3’	NM_199489.4
*CXCL9*	F 5′- AGACCCAGATTCAGCAAGGG -3’ R 5′- TCTTTGACTCCGGATGGTGG-3’	NM_145672.4
*CXCL10*	F 5′- GCTGGTCCGAATCTTCCCTC -3’ R 5′- TTTGCCATCTCACCTGGACT-3’	NM_139089.2
*CHOP*	F 5′- CACAAGCACCTCCCAAAG -3′ R 5’- CCTGCTCCTTCTCCTTCAT -3’	NM_001109986.1
*JNK*	F 5′- AGTGTAGAGTGGATGCATGA -3′ R 5’- ATGTGCTTCCTGTGGTTTAC -3’	NM_053829.2
*EIF-2*	F 5′- CTTTCCGGGACAAGATGGCG -3′ R 5’- CTCTGTGAAGTGTGGGGGTC -3’	NM_001399818.1
*ATF-6*	F 5′- AAGTGAAGAACCATTACTTTATATC -3’ R 5’-TTTCTGCTGGCTATTTGT-3’	NM_001107196.1
*XBP-1*	F 5′- TTACGAGAGAAAACTCATGGGC -3’ R 5’-GGGTCCAACTTGTCCAGAATGC-3’	NM_001004210.2
*BIP*	F 5′- AACCAAGGATGCTGGCACTA -3’ R 5’-ATGACCCGCTGATCAAAGTC -3’	NM_013083.2
*NF-KB*	F 5′-ACAGATATACCACTGTCAACAGCA-3’ R 5’-TTTGCAGGCCCCACATAGTT-3’	XM_039103226.1
Caspase-3	F 5′-GAGCTTGGAACGCGAAGAAA-3′ R 5’-ACACAAGCCCATTTCAGGGT-3’	NM_012922.2
*β-actin*	F 5′-CGTTGACATCCGTAAAGAC-3′ R 5’-TGGAAGGTGGACAGTGAG-3’	NM_031144.3
*GAPDH*	F-TGC TGG TGC TGA GTA TGT CG-3′ R-TTG AGA GCA ATG CCA GCC -3′	NM_017008

[tumor Necrosis Factor alpha (*TNF-α*), interleukin (*IL-17, IL-β*; *IL-10*)], vascular endothelial growth factor (*VEGF*), C-C chemokine receptor type 7 (*CCR7*), *CXC* motif chemokine ligand 9 (*CXCL9*), *CXC* motif chemokine ligand 10 (*CXCL10*), C/EBP homologous protein (*CHOP*), c-Jun N-terminal kinase (*JUK*), eukaryotic initiation factor 2α (eIF2α), activating transcription factor (*ATF-6*), X-box-binding protein-1 (*XBP1*), Immunoglobulin-binding protein (*BiP*), Nuclear Factor Kappa B Subunit (NF-κB), caspase-3. 2.14.

### 2.14. Histopathology determination

The colon specimens of rats from various groups were obtained and promptly preserved in a 10% buffered neutral formalin solution for 48 hours. Subsequently, the specimens were dried using a gradual increase in alcohol concentration, cleaned using xylene, and finally embedded in paraffin wax. The paraffin sections were sliced to a thickness of 5µm using a microtome (Leica RM 2155, England). The sections were appropriately prepared and subjected to routine staining using hematoxylin and eosin stains. Subsequently, a microscopic examination was conducted [47]. The stained sections were subjected to examination in order to identify any potential pathological alterations in the tissues. The grading of histopathological lesions was determined by evaluating the histo-morphological alterations in five areas per section for the colon specimens [48].

### 2.15. Immunohistochemistry assay

The paraffin sections underwent immunostaining using specific antibodies. These included a Recombinant Anti-Beclin 1 Rabbit monoclonal antibody [EPR20473] (ab210498), at 1/100 dilution, Cambridge,UK., abcam., and Recombinant Anti-JNK1 Rabbit monoclonal antibody [EPR17557] (ab199380). Tissue sections measuring 5 μm from all experimental groups were prepared on charged slides, dewaxed, and hydrated. Antigen retrieval was performed according to the manufacturer’s instructions. The slides were subjected to a wash in phosphate-buffered saline (PBS) solution, followed by incubation with the secondary antibody. Specifically, the secondary antibody used was the expose rats and rabbit specific HRP/DAB detection kit from Abcam (Cat. #: ab80436), which was used in its ready-to-use form. This incubation step lasted for 15 minutes and was carried out at room temperature in a chamber with controlled humidity. Prior to this, the sections had undergone overnight incubation at a temperature of 4 °C with the primary antibodies. The specimens were immobilized in a synthetic mounting medium and subsequently stained with hematoxylin as a counterstain. Following this, the specimens were dehydrated and mounted. The staining process involved the use of the DAB chromogenic agent, namely the Expose mouse and rabbit specific HRP/DAB detection kit (abcam; Ready-to-use; Cat. #: ab80436). A total of five animals were utilized for analysis in each group, and for every antigen, three immunolabeled slices were extracted from each animal and subjected to analysis [49].

### 2.16. Statistical analysis

Multiple group comparisons were made using one-way analysis of variance (ANOVA) and Tukey’s post hoc test for statistical significance. The assessment of variance normality and homogeneity was conducted using the Shapiro-Wilk test for normality and Levene’s test for homogeneity, respectively. The level of significance chosen was less than 0.05. The graphs were generated using Graph Pad Prism 5 (Graph Pad Software, La Jolla, CA, USA).

## 3. Results

### 3.1. Assessment of clinical signs related to colonic inflammation.

**Fig 2:**
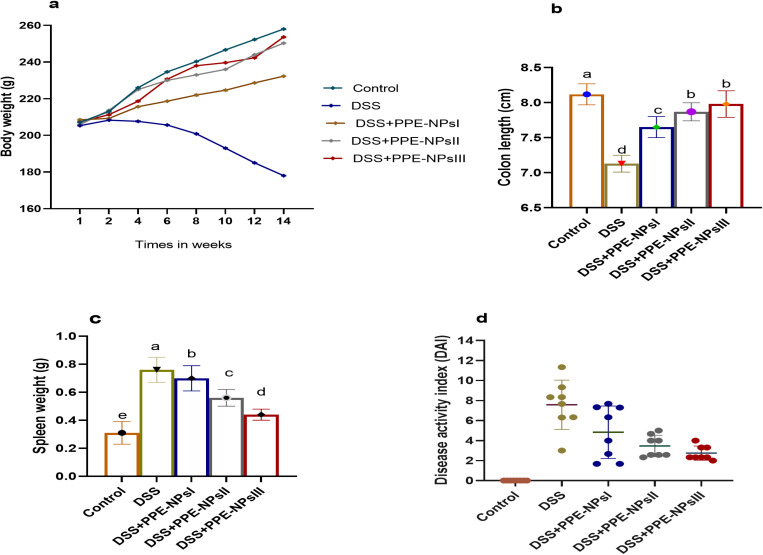
Changes in (a) Body weight gain. (b) Colon length. (c) Spleen weight. (d) Disease activity index score in DSS induced colitic rats in response to pomegranate peel extract loaded nanoparticles (PPE-NPs) treatment. Control: Non-colitic group, Colitic groups include: DSS, PPE-NPsI, PPE-NPsII and PPE-NPsIII groups where rats were orally gavaged with dextran sodium sulphate (DSS) and treated with 0, 50, 100 and 150 of pomegranate peel extract loaded nanoparticles, respectively. Values are expressed as mean±SE. ^a-d^Means of the bars with different letters were significantly different among groups(P < 0.05).

The effectiveness of PPE-NPs in reducing colitis symptoms in rats after induction is shown in Fig 2. The rats with DSS-induced colitis experienced significant weight loss due to inflammation in the colon, whereas the groups treated with PPE-NPs had a more rapid restoration of body weight (Fig 2a). Additionally, the groups of rats treated with PPE-NPs had a substantially longer colon (P < 0.05) compared to the group of rats induced with DSS (Fig 2b). The DSS colitic rats showed a significant increase in spleen size, but the group treated with PPE-loaded CSNPs had a spleen weight that was almost the same as the non-colitic group (Fig 2c). An incremental rise in DAI scores, which are indicative of colon inflammation, was consistently detected in the DSS-induced groups during experiment. Meanwhile, the groups of rats treated with PPE-NPs had lower DAI score in comparison with DSS-induced colitis, which was statistically significant (Fig 2d).

### 3.2 Hematological and biochemical estimation

The impact of PPE-NPs on the hematological, hepatic, and kidney function tests is presented in Table 4. The RBC counts were considerably reduced in rats induced with DSS, with a p-value of less than 0.05. Conversely, colitic rats that received different concentrations of PPE-NPs (with no signfaicane in between) did not exhibit any noteworthy disparities in RBC levels in comparison to non-colitic rats in the control group. Furthermore, the rats in the DSS group exhibited the most significant decrease in Hb concentration (p < 0.05), however this decrease was reversed in the group that received treatment with PPE-NPs. The higher levels of AST and ALT in rats with colitis caused by DSS were significantly decreased in PPE-NPs treated in a dose dependent manner. In addition, the DSS group of rats treated with PPE-loaded CSNPs showed a significant reduction in urea and creatinine levels following colitis induction especially in PPE-NPsII and PPE-NPsIII. Significantly, the ingestion of PPE-NPs during the onset of colitis effectively preserved red blood cell counts and hemoglobin concentrations, comparable to those of the non-colitic group, and restored liver and kidney functions.

**Table 4:** Changes in liver and kidney function tests and hematological indices in DSS induced colitic rats in response to PPE-NPs treatment.

Parameter	Control	DSS	DSS + PPE-NPsI	DSS + PPE-NPsII	DSS + PPE-NPsIII	SEM	*p*-Value
RBCs (×10^6^/ µ L)	12.93^a^	6.93^c^	7.30^b^	10.10^ab^	11.06^ab^	0.11	<0.001
HB (g/L)	11.95^a^	8.03^c^	9.00^b^	10.34^ab^	11.75^ab^	0.14	<0.001
ALT (U/L)	39.58d	225.33^a^	115.51^b^	99.80^c^	45.01^d^	0.23	<0.001
AST(U/L)	22.70^e^	161.42^a^	97.45^b^	64.1o^c^	34.43^d^	0.16	<0.001
Urea (U/L)	32.80^d^	62.90^a^	42.53^b^	39.10^c^	34.52 cd	0.17	<0.001
Creatinine (U/L)	0.75^d^	1.76^a^	1.05^b^	0.98^c^	0.91^c^	0.09	<0.001

PPE-NPs, pomegranate peel extract loaded nanoparticles, ALT, alanine transaminase; AST, aspartate transaminase; RBCs, red blood cells; Ht, hematocrit; Hb, hemoglobin; SEM, standard error of mean. ^a-e^ Means of the rows with different letters were significantly different among groups (*p* < 0.05). Control: Non-colitic group, Colitic groups include: DSS, PPE-NPsI, PPE-NPsII and PPE-NPsIII groups where rats were orally gavaged with dextran sodium sulphate (DSS) and treated with 0, 50, 100 and 150 of pomegranate peel extract loaded nanoparticles, respectively.

### 3.3 Assessment of fecal calprotectin levels

Table 5 depicted the calprotectin levels in fecal samples following the onset of colitis. The levels of fecal calprotectin in the groups with colitis were considerably increased (p < 0.05), whereas in the groups treated with PPE-NPs their levels were decreased especially at higher levels. The significant decrease in fecal calprotectin levels was notably more pronounced in the group treated with PPE-NPsIII (p < 0.05) compared to the group with colitis.

**Table 5:** Changes in fecal calprotectin levels in DSS induced colitic rats in response to PPE-NPs treatment.

Parameter	Time	Control	DSS	DSS + PPE-NPsI	DSS+ PPE-NPsII	DSS + PPE-NPsIII	SEM	*p*-Value
Fecal calprotectin (μg/g feces)	2 dpt	40.00^c^	97.60^a^	81.80^b^	81.20^b^	48.90^c^	0.34	<0.001
	4 dpt	36.83^e^	132.40^a^	121.40^b^	79.40^c^	59.83^d^	0.33	<0.001
	6 dpt	37.30^e^	171.40^a^	136.10^b^	122.40^c^	78.14^d^	0.27	<0.001
	8 dpt	36.10^e^	194.50^a^	149.40^b^	136.80^c^	110.83^d^	0.14	<0.001
	10 dpt	35.83^e^	208.70^a^	160.91^b^	145.60^c^	104.93^d^	0.29	<0.001
	12 dpt	35.32^e^	239.10^a^	116.41^b^	103.40^c^	51.00^d^	0.11	<0.001
	14 dpt	37.60^e^	265.20^a^	102.10^b^	75.50^c^	49.10^d^	0.15	<0.001

pomegranate peel extract loaded nanoparticles, Days post DSS induction: dpi. Control: Non-colitic group, Colitic groups include: DSS, PPE-NPsI, PPE-NPsII and PPE-NPsIII groups where rats were orally gavaged with dextran sodium sulphate (DSS) and treated with 0, 50, 100 and 150 of pomegranate peel extract loaded nanoparticles, respectively. SEM, standard error of means. ^a-e^ Means of the rows with different letters were significantly different among groups (*p* < 0.05). dpt: days post treatment.

### 3.4. Assessment of C-reactive protein levels, myeloperoxidase and nitric oxide in Colonic Tissues

**Fig 3:**
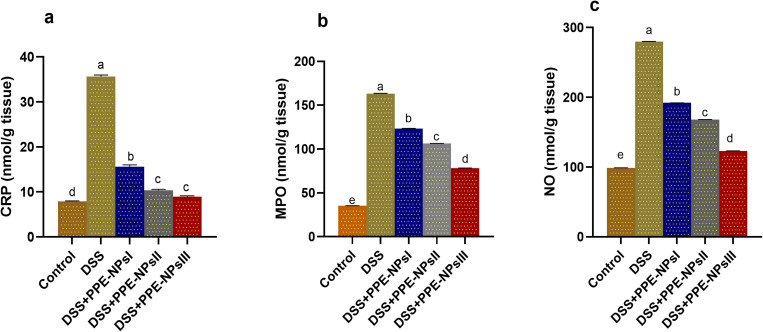
Changes in c-creative protein (CRP) Levels and of myeloperoxidase (MPO) and nitric oxide (NO) in colonic tissues in DSS induced colitic rats in response to pomegranate peel extract loaded nanoparticles (PPE-NPs) treatment. Control: Non-colitic group, Colitic groups include: DSS, PPE-NPsI, PPE-NPsII and PPE-NPsIII groups where rats were orally gavaged with dextran sodium sulphate (DSS) and treated with 0, 50, 100 and 150 of pomegranate peel extract loaded nanoparticles, respectively. Values are expressed as mean ± SE. ^a-d^ Means of the bars with different letters were significantly different among groups (p < 0.05).

The levels of CRP increased following DSS induction. In comparison, the groups that received PPE-NPsII and III showed decreased levels of CRP, respectively, as compared to the colitic group. The colitic groups had the greatest levels of MPO activity in colon tissues, which was significantly reduced in the group treated with different concentrations of PPE-NPs in a dose dependent manner (Fig 3) (p < 0.05). Notably, the induction of DSS increased the levels of NO, while NO recorded lowest (p < 0.05) levels in PPE-NPsIII treated group.

### 3.5. Assessment of inflammatory endoplasmic stressors mediators in colon by Real-Time PCR

**Fig 4:**
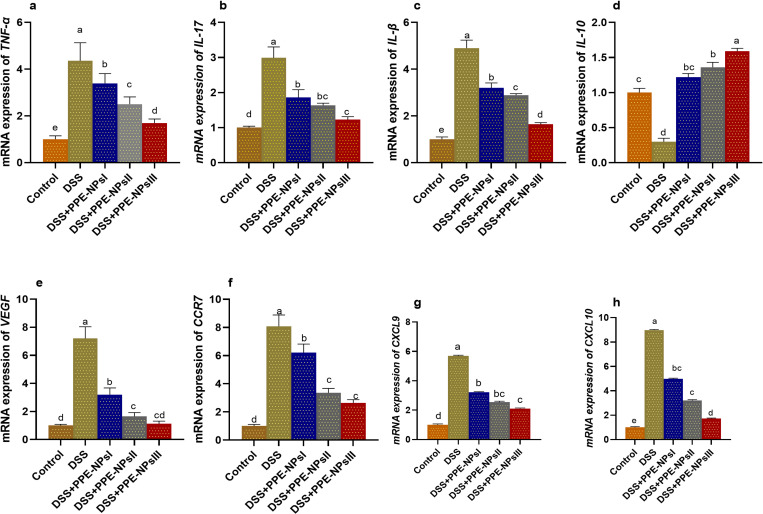
Changes in inflammatory mediators [tumor Necrosis Factor alpha (*TNF-α*, a), interleukin (*IL-17*, b; *IL-β*, c; *IL-10*, d)], vascular endothelial growth factor (*VEGF*, e), C-C chemokine receptor type 7 (CCR7, f), CXC motif chemokine ligand 9 (*CXCL9*, g), *CXC* motif chemokine ligand 10 (*CXCL10*, h) in colonic tissues in DSS induced colitic rats in response to pomegranate peel extract loaded nanoparticles (PPE-NPs) treatment. Control: Non-colitic group, Colitic groups include: DSS, PPE-NPsI, PPE-NPsII and PPE-NPsIII groups where rats were orally gavaged with dextran sodium sulphate (DSS) and treated with 0, 50, 100 and 150 of pomegranate peel extract loaded nanoparticles, respectively. Values are expressed as mean ± SE. ^a-d^ Means of the bars with different letters were significantly different among groups (p < 0.05).

The inflammatory response in the colon was assessed by measuring the levels *of IL-17, TNF*-*α, IL-10* and *IL-1β* cytokines, as shown in Fig 4. The highest inflammatory response (*p* < 0.05) was perceived in the colitic non-treated group, as proved by the remarkable increase in pro-inflammatory cytokines *IL-17*, *TNF-α*, and *IL-1β* levels. Meanwhile, the levels of these inflammatory cytokines were lowered in the groups that received PPE-loaded NPs. The mRNA expression of TNF-α levels were significantly downregulated (*p* < 0.05) with increasing the concentration of PPE-loaded PPE-loaded NPs when compared with colitic non treated group. Groups treated with PPE-NPsII and PPE-NPsIII showed the prominent lower expression levels of *IL-1β* gene*.* The mRNA expression levels of *VEGF* and *CCR7* were detected in groups that received PPE-NPsII and PPE-NPsIII for 14 days treatment. Additionally, higher levels of PPE-NPs markedly reduced *CXCL-9* levels. The most prominent downregulation of *CXCL-10* was noted in group treated with PPE-NPsIII.

**Fig 5:**
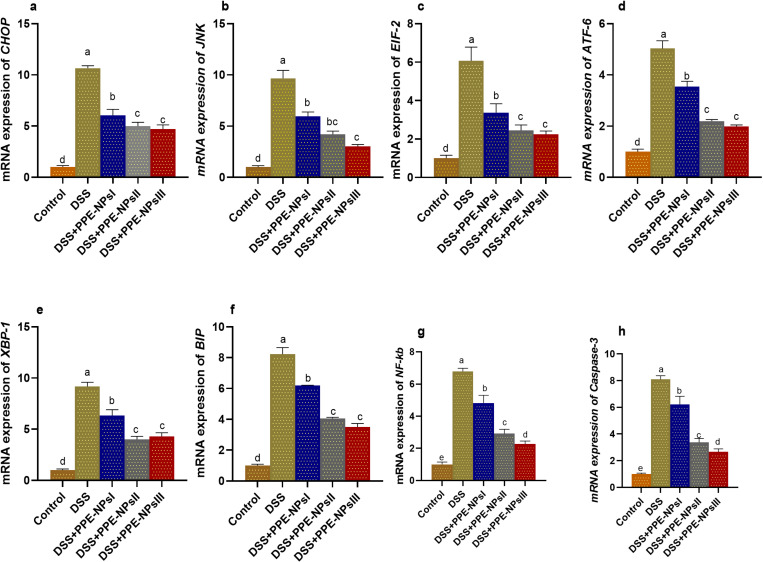
Changes in expression endoplasmic stressors related genes C/EBP homologous protein (*CHOP*, a), c-Jun N-terminal kinase (JUK, b), eukaryotic initiation factor 2α (*eIF2α*, c), activating transcription factor (*ATF-6*, d), X-box-binding protein-1 (*XBP1*, e), Immunoglobulin-binding protein (BiP, f), Nuclear Factor Kappa B Subunit (NF-κB, g), caspase-3 (h) in colonic tissues in DSS induced colitic rats in response to pomegranate peel extract loaded nanoparticles (PPE-NPs) treatment. Control: Non-colitic group, Colitic groups include: DSS, PPE-NPsI, PPE-NPsII and PPE-NPsIII groups where rats were orally gavaged with dextran sodium sulphate (DSS) and treated with 0, 50, 100 and 150 of pomegranate peel extract loaded nanoparticles, respectively. Values are expressed as mean ± SE. ^a-d^ Means of the bars with different letters were significantly different among groups (p < 0.05).

The gene expression response of endoplasmic stressors in the colon was assessed by measuring the levels of *CHOP*, *JNK, EIF-2, ATF-6, XBP-1, BIP, NF-KB*, and caspase-3, as shown in Fig 5. The highest response (*p* < 0.05) was perceived in the colitic non-treated group, as proved by the remarkable increase in genes expression levels. Meanwhile, the levels of these expressions were remarkably lowered in the groups that received PPE-NPs. Notably, *CHOP, EIF-2, ATF-6, XBP-1, BIP* reached their lower levels in groups treated with PPE-NPsII and PPE-NPsIII. The most downregulation of mRNA expression of *NF-KB was detected* PPE-NPsIII received group (decreased to 2.27-fold change). The mRNA expression levels of caspase -3 were dose dependently downregulated in group treated with PPE-NPs in comparison with colitic non treated group (6.21, 3.36 and 2.65 in PPE-NPsI, PPE-NPsII and PPE-NPsIII treated groups, respectively vs 8.09 in DSS group).

### 3.5. Assessment of oxidative stress mediators in colon by enzyme linked immunosorbent assay.

**Fig 6:**
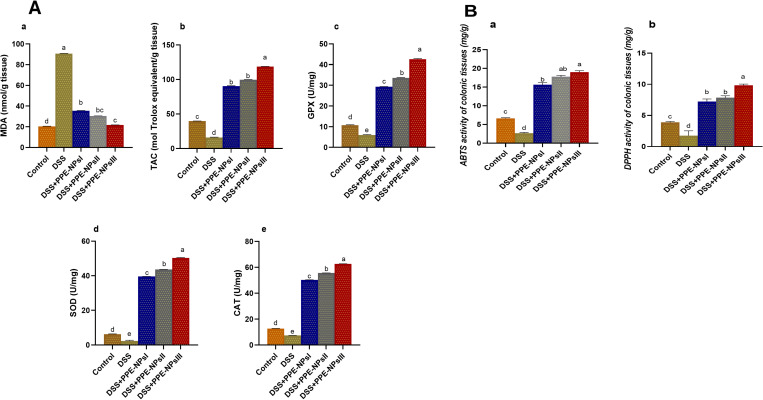
Changes in oxidative stress mediators, Fig 6.A (malonaldehyde (MDA, a) total antioxidant capacity, (TAC, b), glutathione peroxidase (GPX, c), superoxide dismutase (SOD, d), catalase (e) and Fig 6.B described the changes in scavenging capacity in colonic tissues against ABTS (2,2-azinobis-(3-ethylbenzothiazoline-6-sulfonic acid, a) and DPPH (1,1-diphenyl-2- Picrylhydrazyl, b) free radicals in colonic tissues in DSS induced colitic rats in response to pomegranate peel extract loaded nanoparticles (PPE-NPs) treatment. Control: Non-colitic group, Colitic groups include: DSS, PPE-NPsI, PPE-NPsII and PPE-NPsIII groups where rats were orally gavaged with dextran sodium sulphate (DSS) and treated with 0, 50, 100 and 150 of pomegranate peel extract loaded nanoparticles, respectively. Values are expressed as mean ± SE. ^a-d^ Means of the bars with different letters were significantly different among groups (p < 0.05).

In Fig 6. A, the MDA levels increased following DSS induction. In comparison, the groups that received PPE-NPs especially at higher concentrations showed decreased MDA levels, as compared to the colitic group. The group treated with PPE-NPsIII had the greatest levels of TAC in colon tissues, which were significantly reduced in the colitis group (Fig 6. A) (p < 0.05). the higher antioxidant activities of GPX, SOD, CAT were found in groups that received PPE-NPs in a dose related manner unlike DSS untreated group.

### 3.6. Assessment of scavenging activities in colonic tissues

The results of evaluating the impact of different levels of PPE-NPs on scavenging activities were illustrated in Fig 6. B. Colitic groups received higher levels of PPE-NPs showed significant (p < 0.05) strong scavenging ability against synthetic ABTS free radical when compared with DSS non treated group. Moreover, the highest significant (p < 0.05) scavenging ability against DPPH free radical was detected in colitic group received PEE-NPs at the level of 150 mg/kg.

### 3.8. Histopathological Changes in the Colon

**Fig 7:**
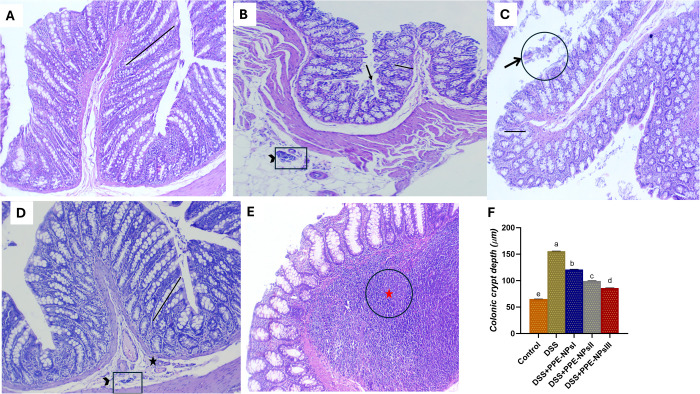
Photomicrograph of colon showing normal histological structures of mucosa with its invaginated crypts, submucosa, musculosa and serosa in control non-colitic group (a). Necrosis of some columnar epithelial lining mucosa (arrow), leukocytic infiltrations within lamina propria, submucosa and impacted serosal blood vessels with round cells (black arrowhead) in DSS induced colitic group (colitic non treated group, b) (H&E 10x). necrotic and denuded some epithelial lining mucosa (arrow) in PPE-NPsI (colitic group treated with pomegranate peel extract loaded nanoparticles at the level of 50 mg/kg, c). H&E 10x. Round cells infiltrations (black arrowhead) and edema (star) within submucosal layer in group treated with PPE-NPsII (100 mg/kg). H&E 10x,40x respectively (e). Normal colonic layers with prominent mucosal lymphoid follicles (red star) extending towards the submucosa in group treated with PPE-NPsIII (150 mg/kg) (d). H&E 40x respectively.

Histopathological assessment described a statistically significant change in colonic tissues among different groups Fig 7. The colonic tissue in DSS induced group that not treated displayed intense inflammation characterized by areas of intense inflammatory infiltrate of polymorphonuclear cells, in addition to necrosis of some columnar epithelial lining mucosa, leukocytic infiltrations within lamina propria, submucosa(a). serosal blood vessels were impacted with round cells (b). Notably, the severity of histopathological lesions was reduced in groups treated with PPE-NPs in a dose dependent manner. In was cleared that, colonic tissues in group treated with PPE-NPsI at the level of 50 mg/kg of colon showed necrotic and denuded some epithelial lining mucosa (c) whereas, PEE-NPsII treated with PPE-NPs at the level of 100 mg/kg showed apparently normal intestinal layers with some round cells infiltrations and edema were seen within submucosal layer in some examined sections (d). Remarkably, treatment with higher level of PPE-NPsIII (150 mg/kg) exhibited normal colonic layers with prominent regeneration with large mucosal lymphoid follicles which extend towards the submucosa (e).

### 3.9. Immunohistochemistry assay

**Fig 8:**
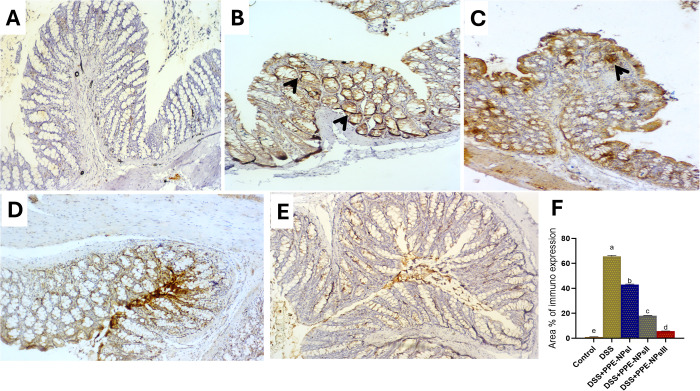
Representative photomicrograph of Beclin-2-immunohistochemical staining sections of rat’s colon showing unmarked immunoreactive cells in the control non colitic group (a). Overexpression of positive immune cells were found in colitic non treated group (b). Reduction of beclin immunostained cells in colitis group treated with PPE-NPsI (pomegranate peel extract nanoparticles, 150 mg/kg) (c). Moderately detected immune-reactive cells were detected in a group treated with PPE-NPsII (100 mg/kg, d). Mild immunostained cells in colitis group treated with PPE-NPsIII (150 mg/kg, e). The arrowheads demonstrated positive cells. IHC counterstaining with Mayer’s haematoxylin. Scale bar 100 µm.

**Fig 9:**
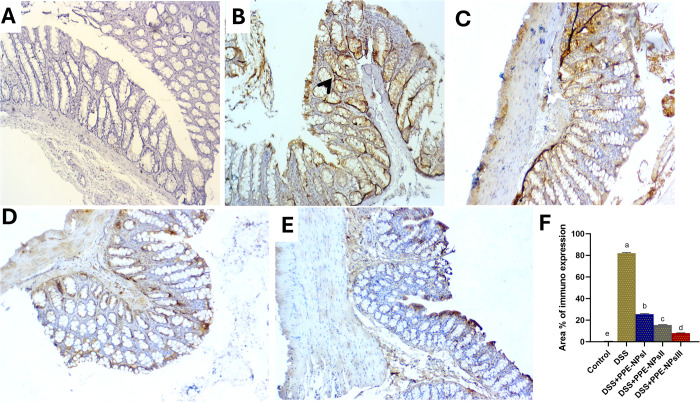
Representative photomicrograph of JUK-immunohistochemical staining sections of rats colon showing undetectable stained cells in control non-colitic group (a), strongly expressed immune cells were observed in colitic non-treated group (b), barely expressed cells were detected in response to treatment with PPE-NPsI (50 mg/kg, c), slightly lower expression were described in treatment with PPE-NPsII (100 mg/kg, d), and moderately expressed cells were noted in treatment with PPE-NPsIII (150 mg/kg, e).The arrowheads demonstrate the positive cells. IHC counterstaining with Mayer’s haematoxylin. Scale bar 100 µm.

The expression of beclin-2 was described in Fig 8. In the non-colitic group, revealed hardly undetectable stained cells (a). Remarkably, colitic group that did not receive any treatment exhibited strongly over expressed stained cells against beclin-2 in colonic mucosa and submucosa (b). Moderate expression level for beclin-2 was seen in group treated with PPE-NPsI (c). Moreover, the expression of beclin-2 was slightly lower in the group treated with PPE-NPsIII (e) than group treated with PPE-NPs II (d) in both submucosa and lamina propria. Regarding the expression of Junk Fig 9, undetectable staining for junk was found in non-colitic group (a). Meanwhile strong immunoreactivity levels against Junk were markedly demonstrated in the colitic non treated group (b) Obviously, colitis groups treated with PPE-NPs showed a reduced expression level of Junk. PPE-NPsI had a decreased expression of immune-reactive cells than colitic group non treated group (c). Moderate to minor positive cells were seen in PPE-NPsII and III treated groups, respectively (d and e).

## 4. Discussion

Dietary polyphenols exist in Punica granatum (pomegranate), have shown to exert strong anti-inflammatory and antioxidant properties and it has been reported that using of pomegranate extract has been displayed as effective alternative therapies to ameliorate colonic inflammation [50,51]. However, these active components that present in the pomegranate extract, especially those from peels, are highly sensitive to environmental conditions which adversely affect them and weaken their functions [52]. Recently encapsulation of pomegranate peel’s polyphenolics in a nanoscale particulates has been studied extensively to trigger their bio availability, solubility, accessibility and storage stability [53]. Nevertheless, their efficacy to enhance the conventional therapeutic strategies on the management of IBD have not been fully understood until now at lower doses in comparison with free form. In DSS induced colitic and non- treated rats notable elevated DAI scores were detected as assessed by body weight loss that resulted from malabsorption of nutrient and diarrhea that be associated with by colorectal bleeding were detected. Additionally, a shorten in colon length with denoted spleen enlargement was detected in the colitic non-treated group. Similarly, DSS-induced mice exhibited, identifiable weight loss (about 5–10% decline) and altered stool consistency as a result of bloody diarrhea [54]. In accordance, our results described that higher levels of PPE-NPs could reduce the severity of colonic damage induced by DSS in rats as reflected by improvement of DAI score and recovery of weight loss and colon length reduction. Similarly, polyphenolic compounds attenuated colitis severity and its associated bad clinical observations [55]. Furthermore, nano-encapsulation using natural-based nanoparticles protected the probiotic against harsh gastric conditions and helped in retaining its bioactive properties during the in vitro digestion process and subsequently improved gut health [56]. Moreover, pomegranate peel polyphenols reduced chronic gut inflammatory responses damage in rats [57,58]. Also, polyphenols alleviated the severity of IBD clinical symptoms in mice including excessive diarrhea, and an elevated of body weight loss [59]. Also, abundant productions of reactive oxygen metabolites have been shown to be account for the electrolytes secretion and water with consequence of diarrhea however, treatment with ellagic acid enriched pomegranate peel extract could decrease the adverse impacts associated with oxidative stress [60]. In addition, weight loss and incidence of intestinal adhesions were decreased and associated with lessening in chronic colonic inflammation level after feeding on ellagic acid-enriched pomegranate extract [50]. The alleviated impact of PPE-NPs was developed from encapsulation of their polyphenolic compounds in nano carrier that prolonged retention and delivery of the polyphenol. In accordance, nanoparticles contained curcumin improved mucus penetration and ROS-responsive drug release functions for the curcumin delivery to colitis tissues, accordingly, resulted in more efficient IBD therapy [61,62]. Ultimately promoting a higher efficacy of PPE-NPs in colitis therapy than free form of pomegranate extract in previous study was attributed to enhancing pomegranate bioactive components stability as well as its bioavailability by their incorporation into a nano carrier [63]. Moreover, nanoencapsulation of pomegranate peel extract provides protection of their compounds against the aggressive environmental factors, release of these active compounds at controlled rates at targeted place under specified conditions, and improving nutritional value [64].

Herein, rats in DSS model of colitis that non treated displayed higher levels of NO than PPE-NPS treated groups. Concurrently generation of small quantities of NO display anti-inflammatory action inside endothelial cells while, along the course of colon inflammation the enzyme iNOS yields large NO amounts which contributes to the excessive inflammation response [65]. Similarly, Tugcu et al. [66] showed a decrease in NO levels in rats with nephrolithiasis fed pomegranate juice [66].

Moreover, the inflammation developmental process of promoted disease agent can trigger the release of essential cytokines, which all account for CRP induction [67]. Furthermore, colitis severity is accompanying higher levels of CRP, common pro-inflammatory agent, which is a biomarker of colon inflammation [68]. Moreover, MPO activity is a an indicative biomarker of leucocyte infiltration and evidenced for inflammatory reactions in many tissues [69]. In the current study, the influx of inflammatory cells into the injured colon was prominent in the colitic group and was accompanied by increased colonic MPO activity, which is in accordance with Ali et al.’s results [50,70]. This study demonstrated that the damage related to colitis was associated with a significant increased levels of CRP and MPO activities in the colon. Interestingly these higher levels of CRP and MPO were remarkably diminished in those rats that received higher PPE-NPS levels, which supported their protecting impact against colon damage [50]. Additionally, higher fecal calprotectin levels is an indicator of increased disease activity in colitis [71]. The higher fecal calprotectin involved in migration of neutrophil into the intestinal mucosa because of intestinal inflammation [72]. Lowering the level of fecal calprotectin in groups treated with PPE-NPS proved their role in subsiding inflammatory response[58]. The efficient systemic administration, stability, and higher bioavailability of PPE-NPS in the current study could effectively reduce the higher inflammatory response concurrent with induced colitis [73]. Several biomarkers correlated to inflammation, and angiogenesis, have been conveyed to be increased in the serum and intestinal mucosa of patients harboring IBD. Elevated levels of these markers may result from microvasculature changing in the inflamed intestine, and ongoing angiogenesis may provoke the inflammatory process, promoting to the pathology of IBD [74]. As leukocytes at inflammation site generate mediators like vascular endothelial growth factor (VEGF), an angiogenic cytokine that trigger vascular permeability and potentiate blood growth capillary [75]. Herein, induction of colitis by DSS provoked an overexpression levels of *VEGF* which in the same line of [76] while treatment with PPE-NPs downregulating their expression levels. In accordance, polyphenols can reduce inflammatory angiogenesis by suppressing VEGF-induced oxidative stress and NF-κB signaling [77,78].

It has been documented that that prolonged oxidative stress plays a key role in the initiation and progression of IBD [79]. Several studies have stated that IBD is accompanying with ROS over production or reduced antioxidant activity with subsequent oxidative stress due to an imbalance between antioxidant activity and ROS [80,81]. In the current study, DSS induced colitic rats exhibited higher levels of oxidation products (MDA) with lower TAC and antioxidant enzymes activities and these findings agreed with [82] indicated that colonic inflammation was associated with destruction of the endogenous antioxidant system and lipid oxidation in the intestinal tissues. In accordance, feeding on polyphenolic compounds in pomegranate peel produced the most effective protective impacts against oxidized fish oils -induced oxidative injury in the intestinal tissues [83]. Consistent with our findings, therapeutic approach based on pomegranate peel extract reduced the oxidative stress accompanied intestinal inflammation. The potent anti-oxidative role of PPE in colitic rats was supported by reducing MDA, oxidative stress marker, and increasing TAC via its detoxification function and modifying MDA formation in the intestinal mucosa [84]. Moreover, the intrinsic antioxidant properties of polyphenolics exists in PPE have been ascribed due to its free radical scavenging activity and thus protected against free radical-induced damage [85]. Moreover, these outcomes were also, supported by significant antioxidant activity of higher levels of PPE -NPs via scavenging synthetic DPPH and ABTS free radicals which came in accordance with [86]. Similarly, plant rich in flavoindes such as ginkgo biloba leaves can regulate redox homeostasis inside the body [87]. The protective antioxidant potential properties of the PEE-NPs in this study evinced by their ability to prevent ROS generation in the colonic tissues via scavenging DPPH and ABTs free radicals’ formation [88]. Recently, developing of nano carrier as delivery systems for PPE successively have been potentiated its antioxidant activity via enhancing its solubility and concentration in targeted site [89]. Moreover, Encapsulation of polyphenolics bioactive materials in PPE by nano envelope could protect them from adverse environmental effects, and therefore, the prolong the shelf life these unstable compounds, developing targeted-delivery, controlled- and effective-release and thus achieve a prolonged therapeutic and functional effects [90].

Inflammation has a key role in IBS pathogenesis which can worsen the symptoms via triggering the visceral sensory system and the expression of pro-inflammatory mediators including cytokines [91]. The intestinal immune system that is experiencing inflammation exhibits an increased concentration of pro-inflammatory cytokines, and chemokines and while the anti-inflammatory cytokines that promote resolution are insufficiently represented. This imbalance frequently leads to persistent inflammation and the development of conditions such as IBD [2,92].

Multiple investigations have shown that both chronic unresolved and acute inflammation of the gastrointestinal tract result in an imbalance in the inflammatory cytokines profile [1] which also, agreed with our outcomes in colitic non-treated group. The NF-κβ pathway has a pivotal role in the inflammatory response, particularly in controlling the expression of cytokines implicated in inflammation [93,94]. It was established that polyphenols can inhibit inflammatory responses by suppressing NF-κβ and mitogen-activated protein kinase signaling pathways [95]. Additionally, *Cxcl 9–10* were highly upregulated in the colon of colitis model [96] and IL-17 is thought to contribute to the IBD development [97]. Also, increased levels of IL-6 in both the blood serum and tissue samples from the mucosa are a reliable marker for IBD [98]. In the era of potential immunoinflammatory therapeutic strategies, several experimental studies support a beneficial role of dietary polyphenols, secondary plant metabolites, ubiquitously present in fruits and vegetables, in several inflammatory diseases, including IBD [50,99]. Our findings afford support for anti-oxidative and anti-inflammatory properties of PPE-NPs based on downregulating the expression of investigated inflammatory mediators. Similarly, Rosillo et al. [50] explored the ellagic acid enriched pomegranate extract effects on the chronic Crohn’s disease model in mice. The enhanced immune response in group treated with PPE-NPs can attributed their mechanistic impact comprising an enhanced total antioxidant activity and reduced molecular degradation resulted from oxidative stress [100]. Additionally, PPE can alleviate inflammatory symptoms of colitis and reduced serum levels of the inflammatory markers (TNF-α, IL-6, and CRP) owing to its anti-inflammatory properties [60]. In a prior work [101], punicalagin in PPE was found to decrease the phosphorylation of IκBα and p65, hence preventing the NF-κβ pathway activation induced by lipopolysaccharides which accounted for its anti-inflammatory role [102]. Moreover, a study agreed with our work assessed the anti-inflammatory effects of PPE using both an in vitro Caco-2 cell model and an ex vivo pig colonic explant model [102]. Subsequent tests revealed that the elevated levels of PPE demonstrated potent anti-inflammatory properties in biological system which supported by the greatest inhibition of pro-inflammatory cytokine (IL1A, IL6, and CXCL10) expression in the ex vivo model [102]. Additionally, nanoencapsulation of pomegranate extract increased its stability and Potential anti-inflammatory effect [103]. Polyphenol-rich plants have demonstrated efficacy in alleviating symptoms in individuals with IBD during clinical trials [104]. Additionally, the histopathologic features of representative colonic tissue in groups treated with higher levels of PP-NPs showed a higher degree of regeneration after induction of colitis. These finding came in accordance of [105] who describes that the histopathological findings of inflamed colonic tissues was reduced treatment with 200 and 300 mg/kg of phenolic-rich extract from Nopalea cochenillifera. These authors suggested that treatment with such polyphenols contributed to lessen inflammation and enhance regeneration of the intestinal mucosa. Also, bryophyllum pinnatum extract rich in polyphenols improved the cytoarchitecture of the colonic tissue [106]. The augmented properties of PPE after encapsulation by nano particles could be attributed to protecting its sensitive constituents and controlling its delivery and release (Soltanzadeh et al., 2021). ER stress signifies a new pathway that engages the intestinal epithelium, and respective reports propose that the IBD is linked to an initiation of the ER stress [107]. Shkoda et al[108] implied that ER stress strikes secondary to an inflammatory insult to intestinal epithelial cells, also, the impairment of the ER stress response may be as a cause, rather than a consequence, of intestinal inflammation initiates the development of apoptosis cell death [109]. Unresolved ER stress is one of the primary contributors to the pathogenesis of inflammatory bowel diseases (IBD). Sustained and severe ER stress throughout the course of colitis can trigger the production of pro-inflammatory cytokines, leading to intestinal inflammation and persistent disruption of the intestinal mucosal barrier, ultimately resulting in UC inflammation [110]. Numerous studies have shown that ER stress-induced autophagy, cellular self-degradation that plays a vital role in the pathogenesis of colitis [111]. Moreover, impaired autophagy leads to inflammation, intestinal barrier disruption, and an imbalance in intestinal homeostasis, thereby increasing the risk of colonic diseases [112]. The elevated levels of pro-inflammatory cytokines observed in Crohn’s disease (CD) patients have also been linked to autophagy dysregulation [113] as excessive autophagy can lead to uncontrolled inflammation, cell death, and tissue injury [114]. Herein, excessive immunohistochemical staining of Beclin1 in colonic tissues, the key factor in autophagy initiation, [112], was detected in colitic non-treated group. Meanwhile, controlled the autophagy (moderate autophagy) was evidenced in group treated with higher levels of PPE-NPs via controlling Beclin1 expression to the level that inhabit ER stress which came in agreement with Kökten, Gibot [115]. In the same vein, in DSS-induced mice, treatment with curcumin-enriched polyphenolics ameliorated colitis by impeding excessive autophagy and reducing the mRNA and protein expression of autophagy-related 5, LC3-phosphatidylethanolamine conjugate, and beclin-1 in colonic tissue [116]. Additionally, resveratrol is able to modulate autophagy in colitis by reducing expression of its mediators including beclin-1 [116]. It was reported that ER stress markers (*CHOP*, *ATF6*, *JNK* and *BiP* expression) increased significantly in TNBS-inflamed colon [117]. CHOP is involved in ER stress-induced apoptosis through various mechanisms such as down-regulation of Bcl-2 and translocation of Bax to mitochondria [118]. On the other hand, downregulation of CHOP activity compromises cell viability as it is implicated in programmed cell death in response to impaired function of the endoplasmic reticulum, and cells lacking CHOP are significantly protected from the lethal ER stress [119]. BiP has also demonstrated its role in ER stress-mediated apoptosis both in in vivo and in vitro studies [120]. ATF6 expression can boost the expression of inflammatory cytokines and provoke intestinal inflammation by triggering the NF-κB signaling pathway regulating inflammatory gene expression, which aggravate the development of colitis [121]. The transcription factor XBP1, a key factor ER stress response, is needed for progress and maintenance of secretory cells [122]. Also, induction of ER stress in intestinal epithelium through tissue (and cell type)-specific disruption of XBP1 results in spontaneous enteritis due to inability of XBP1-deficient IECs to properly generate antimicrobial activity and respond appropriately to inflammatory signals in the local milieu [122]. In the current study, interplay between ER stress and inflammatory pathways in intestinal epithelial cells was cleared in DSS induced colitic group as supported by increasing inflammatory levels together with higher expression of ER stress genes. Inversely, treatment with PPE-NPs mimics excessive inflammatory response concomitantly with downregulated expression of *CHOP*, *ATF6*, *JNK*, *ATF6* and *BiP*. It is worth noting that, using of PPE-NPs create homeostatic balance by suppressing excess immune activation with consequences with over production of cytokines and also regulate endoplasmic reticulum functions. In this context, natural polyphenols inhibit CHOP up-regulation as markers of ER stress [123]. Additionally, plant-based active ingredient berberine has long been identified to alleviate ER stress response to reduce inflammation and apoptosis in DSS-induced colitis mice model [124].

## 5. Conclusion

The long-term effectiveness of nanotherapeutic approaches with antioxidant properties opens new avenues for the successful treatment of IBD. The results of this study demonstrated that PPE-NPs have a superior efficacy in mitigating DSS-induced colitis in an experimental model. The beneficial effects of PPE-NPs may be attributed to their integration into the nano-delivery system, which provides protection and facilitates precise delivery, enhancing their long-term efficacy. Furthermore, higher levels of PPE-NPs have a greater impact on enhancing the antioxidant status of colitic rats and regulating excessive inflammatory responses and associated damage. Besides their mechanistic role in inhibiting the excessive ER stress response associated with oxidative stress during IBD progression. In conclusion, this study provides valuable insights that enhance our understanding of the potential benefits of using recently formulated PPE-NPs to reduce the severity of the DSS-induced colitis model.

## Supporting information

S1 Data Biochemical data. (XLSX)
